# Is switching to an oral antibiotic regimen safe after 2 weeks of intravenous treatment for primary bacterial vertebral osteomyelitis?

**DOI:** 10.1186/1471-2334-14-226

**Published:** 2014-04-27

**Authors:** Baharak Babouee Flury, Luigia Elzi, Marko Kolbe, Reno Frei, Maja Weisser, Stefan Schären, Andreas F Widmer, Manuel Battegay

**Affiliations:** 1 Division of Infectious Diseases and Hospital Epidemiology, University Hospital, Petersgraben 4, Basel 4031, Switzerland; 2 Medical Faculty, University of Basel, Basel, Switzerland; 3 Division of Clinical Microbiology, University Hospital Basel, Basel, Switzerland; 4 Department of Orthopedic Surgery, University Hospital of University Basel, Basel, Switzerland

**Keywords:** Vertebral osteomyelitis, Switch to oral therapy, Antibiotic management

## Abstract

**Background:**

Vertebral osteomyelitis (VO) may lead to disabling neurologic complications. Little evidence exists on optimal antibiotic management.

**Methods:**

All patients with primary, non-implant VO, admitted from 2000–2010 were retrospectively analyzed. Patients with endocarditis, immunodeficiency, vertebral implants and surgical site infection following spine surgery were excluded. Persistence of clinical or laboratory signs of inflammation at 1 year were defined as treatment failure. Logistic regression was used to estimate the odds ratios (OR) of switch to an oral regimen after 2 weeks.

**Results:**

Median antibiotic treatment was 8.1 weeks in 61 identified patients. Switch to oral antibiotics was performed in 72% of patients after a median intravenous therapy of 2.7 weeks. Switch to oral therapy was already performed after two weeks in 34% of the patients. A lower CRP at 2 weeks was the only independent predictor for switch to oral therapy (OR 0.7, 95% confidence interval 0.5-0.9, p = 0.041, per 10 mg/l increase). *Staphylococcus aureus* was the most frequently isolated microorganism (21%). Indications for surgery, other than biopsy, included debridement with drainage of epidural or paravertebral abscess (26 patients; 42%), and CT - guided drainage (3 patients).

During the follow-up, no recurrences were observed but 2 patients died of other reasons than VO, i.e. the 1 year intention to treat success rate was 97%.

**Conclusions:**

Cure rates for non-implant VO were very high with partly short intravenous and overall antibiotic therapy. Switching to an oral antibiotic regimen after two weeks intravenous treatment may be safe, provided that CRP has decreased and epidural or paravertebral abscesses of significant size have been drained.

## Background

The incidence of vertebral osteomyelitis (VO) is increasing, primarily affecting elderly patients and those with comorbidities [1,2]. Complications of VO, i.e. epidural, paravertebral or psoas abscesses, which result from direct seeding of the microorganism in different compartments [1], may lead to longer hospital stay and higher mortality. To date, there are no consistent data from randomized controlled trials to guide the optimal duration and appropriate route of antibiotic therapy. Recommendations for the length of antibiotic therapy vary considerably [3-5], with suggestion of parenteral treatment between 3 and 8 weeks [6-9]. The mean treatment duration in a French multicentre observational prospective study, involving 110 patients, was 14.7 weeks, with minimum length ranging from 6 to 12 weeks according to the treating centre [6]. Other observational studies show distinctive differences in recurrence rates for treatment duration of less than 4 weeks (>14%), 6 weeks (10%), 8 weeks (>15%) compared to longer than 12 weeks (3.9%) [10].

We retrospectively investigated patients with primary VO in a tertiary care hospital during a 10-year period with the aim to assess predictors of switching from intravenous to an oral antibiotic regimen and to investigate the clinical outcome dependent on different treatment durations.

## Methods

### Study population and design

This study is a retrospective analysis of all patients diagnosed with VO, seen by an Infectious Disease specialist, at the University Hospital of Basel, Switzerland, between 2000 and 2010. The University Hospital of Basel is a tertiary 800-bed institution serving the northwestern part of Switzerland with a population of approximately half a million people.

Patients were selected from the Infectious Diseases patient database. Supplementary data collected by chart review included demographic characteristics, date of hospitalisation, co-morbidities (diabetes mellitus, cardiovascular disease, immunosuppression and renal insufficiency). Blood tests included creatinine, white blood cell count and C-reactive protein (CRP). Further data comprised the clinical presentation of VO such as back pain, fever, neurologic deficits at presentation (i.e. limb weakness, dysesthesia or sensory loss, retention of urine and radiculopathy), the presence of endocarditis, radiological images, microbiological tests (blood cultures, bone biopsies), antibiotic treatment regimen including route of administration (oral or intravenous) and duration and surgical procedures.

Diagnosis of VO was made if two of the following criteria were present: (1) clinical presentation compatible with VO [1] such as fever, back pain or neurological deficits, (2) compatible radiological images [11,12] and (3) identification [13] of pathogen by blood culture, needle biopsy or surgical biopsy. We excluded patients with endocarditis, as the recommended intravenous treatment is at least 4 weeks, surgical site infection following spine surgery because a 2-week intravenous antibiotic course after extensive debridement is generally considered to be adequate before switching to an oral regimen [14,15] and spinal implants or tuberculous VO because of the longer treatment duration [16,17].

### Outcome measures

The physicians, caring for patients after discharge from hospital, were requested to provide follow-up data on the clinical course, laboratory parameters and treatment at 4 and 6 weeks and 1 year after diagnosis of VO.

Recovery was defined as disappearance of all clinical signs and symptoms of VO with no residual disability [18].

Treatment failure was defined as the persistence of clinical signs and symptoms of VO, i.e. fever, residual pain or neurological symptoms, or laboratory signs of inflammation with no other explanation, VO-related re-hospitalization, relapse or VO-related death. The outcome was evaluated after completing the prescribed antibiotic therapy and at 1 year by in-hospital data and a questionnaire sent to the treating physicians.

### Statistical analysis

Basic demographic characteristics, co-morbidities, laboratory and microbiological parameters, surgical and antibiotic management were compared using the _*X*_^2^ test or Fisher’s exact test for categorical variables and the Mann–Whitney test for continuous variables. Logistic regression was used to estimate the odds ratios (OR) of switch to an oral regimen after 2 weeks.

All analyses were performed using STATA™ software version 11 for Windows (StataCorp, College Station, TX, USA). This study was approved by the local Ethics Committee (Ethische Komission beider Basel).

## Results

### Study population

Among 129 patients with VO, 68 patients were excluded because of spinal implants (25), endocarditis (19), surgical site infections (10) and missing follow-up data (14). The final analysis was performed on 61 patients. The median age was 65 years [interquartile range (IQR) 51–76] and most patients were male (61%). Baseline characteristics are shown in Table 1.

**Table 1:** Baseline characteristics of the study population (n = 61)

**Characteristic**		**All patients**	
		n*	%*
Males		37	61
Median age, IQR		65	51–76
Median BMI, IQR		25	21–28
Arterial hypertension		24	39
Diabetes mellitus		7	11
Coronary heart disease		15	25
Renal impairment (clearance <80 ml/min.)		5	8
Fever (>38°C)		17	28
Back pain		57	93
Neurologic al deficits		10	16
Epidural abscess		31	51
Median leucocytes count, IQR (10^9^/l)		10.1	8.0–12.5
Median C-reactive protein (mg/l)		100	62–186
Microorganism	*Staphylococcus aureus*	11	21
	Coagulase negative staphylococci	9	17
	Streptococci spp.	12	20
	*Escherichia coli*	6	11
	Klebsiella spp.	2	4
	*Pseudomonas aeruginosa*	7	13
	*Propionibacterium acnes*	3	6
	Other or unknown	11	21

*unless otherwise stated.

### Clinical presentation and diagnosis of VO

Twenty-seven patients had no comorbidities, all other patients were reported to have more than 1 underlying medical disease, arterial hypertension being the most frequent (39%) followed by coronary heart disease (25%), diabetes mellitus (11%) and renal impairment, i.e. creatinine clearance <80 ml/min (8%). Back pain was by far the most common presenting symptom (93%), followed by fever (28%). Neurologic deficits at presentation were reported in 16% and abscess formation (i.e. epidural or paravertebral) was found in 51% of patients.

*Staphylococcus aureus* and coagulase-negative staphylococci were the most frequently isolated microorganisms, i.e. 21% and 17% respectively, followed by gram-negative bacteria (28%), streptococci (20%) and *Propionibacterium acnes* (5%). In 8 patients no causing microorganism could be identified.

Diagnostic tools for bacteriologic diagnosis included blood cultures, computerized tomography (CT) - guided needle biopsy and open biopsy. In 49 patients blood cultures were performed. Of these, 25 (51%) were positive with microorganisms regarded as causative pathogens for the VO. Biopsies (CT-guided and open) were performed in 50 patients (82%) yielding a causative bacterium in 92%.

Radiological techniques for the diagnosis of VO included computerized tomography (CT) with contrast medium in 10 patients (16%) and/or magnetic resonance tomography (MRI) in 58 patients (95%).

### Management of vertebral osteomyelitis

All patients received empirical antibiotic treatment for VO according to internal guidelines, after diagnostic procedures have been performed being amoxicillin-clavulanate and ceftriaxone the most frequently used (Table 2). The empiric antibiotic therapy was retrospectively considered to be adequate in 47 patients (83%) according to definite microbiological results. The median duration of antibiotic treatment was 57 days (8.1 weeks; IQR 44–83 days). Switch to an oral antibiotic regimen was performed in 72% of patients after a median intravenous therapy of 19 days (2.7 weeks; IQR 14–27 days). In 21 patients (34%) the switch to oral therapy was performed after 2 weeks of intravenous treatment. The most frequently used oral antibiotic treatment was ciprofloxacin or another quinolone alone (35%) and a combination of ciprofloxacin and rifampin (26%).

**Table 2:** Antibiotic treatment and surgical management of 61 patients with primary spondylodiscitis

**Characteristic**		**All patients**	
		n*	%*
Procedure	Surgery	26	43
	CT-guided drainage	3	5
Adequate empirical antibiotic therapy		47	83
Empirical therapy	Amoxicillin-clavulanate	29	48
	Flucloxacillin	5	8
	Ceftriaxon	9	15
	Piperacillin-tazobactam	6	10
	Other beta-lactame antibiotics	5	8
	Ciprofloxacin	5	8
	Other	2	3
Switch to oral antibiotic treatment		43	72
Oral regimen	Ciprofloxacin or other chinolone alone	15	35
	Ciprofloxacin and clindamycin	5	12
	Ciprofloxacin and rifampicin	11	26
	Clindamycin alone	3	7
	Other	9	21
Antibiotic regimen containing rifampicin		15	25
Median duration of total antibiotic therapy, IQR (days)		57	44–83

*unless otherwise stated.

Indications for surgery, other than biopsy, included debridement with drainage of the abscess in 26 of 31 patients with epidural or paravertebral abscess, whereas CT - guided drainage of the abscess was performed in 3 patients. In 2 patients no drainage or surgery was performed due to the very small size of epidural abscess.

### Predictors of switch to an oral antibiotic regimen after 2 weeks

A univariate and multivariate analysis was performed. After adjustment for age, gender, comorbidity, fever and neurological deficits at diagnosis, microorganisms, abscesses (i.e. epidural or paravertebral), surgery and laboratory parameters, correct empirical antibiotic therapy, as well as antibiotic regimen containing rifampicin, lower CRP at 2 weeks compared to baseline CRP was the only independent predictor of switching to an oral antibiotic regimen after 2 weeks (OR 0.7, 95% CI 0.5-0.9, p = 0.041, per 10 mg/l increase).

### Outcome

A complete follow-up was available for 61 patients. During the follow-up, 2 patients died of metastasizing colorectal and bronchial carcinoma, 47 and 51 days after diagnosis of VO, i.e. the 1-year success rate in an intention to treat analysis was 97% and there were no reported re-hospitalisations and/or treatment failures because of VO.

## Discussion

Our study, involving 61 immunocompentent patients with primary vertebral osteomyelitis, illustrates that switching to an oral antibiotic regimen after 2 weeks of intravenous therapy may be safe, provided that symptoms have improved, epidural or paravertebral abscess has been drained and C-reactive protein levels have decreased. Importantly, our results do not extend to patients with endocarditis, surgical site infection and vertebral implants.

The duration of intravenous therapy has not been established so far. Several studies described successful switch to oral antibiotics after 10 days, using oral agents with a high bio-availability and tissue penetration, i.e. fluorquinolones, rifampin, fusidic acid and clindamycin [19], after endocarditis had been excluded [2,20]. In the study of Beronius et al. [20], however, the median duration of oral antibiotic therapy after a short parenteral therapy was 179 days (range 46–640 days), which is much longer than the therapy duration in our patient collective. This might be due to the fact that 27% of the patients in the above mentioned study had tuberculous VO.

Thirty-five percent of our patients were treated with fluorquinolones alone and 26% with a combination of a fluorquinolone and rifampin, all with good outcome, i.e. cure. Fluorquinolones are bactericidal drugs and thereby allow an early switch to the oral route. In a randomized clinical study, the combination of an oral fluorquinolone and rifampin in case of staphylococcal bone or joint infections, resulted in cure rates that were similar to those with the standard intravenous therapy [21].

In our study, a lower CRP at 2 weeks was the only independent predictor of switching to an oral antibiotic regimen. Serum CRP level is closely related with the clinical response to therapy and is therefore the preferred marker of the course of infection [22]. Criteria for discontinuation of antimicrobial therapy include resolution of clinical symptoms as well as normalization of CRP [6,18]. It has been proposed that a weekly decrease in CRP by 50% represents treatment response [23]. Lack of improvement in symptoms such as continued fever and no reduction in pain or a persistently elevated CRP above 30 mg/l are predictors of treatment failure [22,24]. In our study, the early switch to oral antibiotic therapy, i.e. after two weeks, if above mentioned criteria are met, does not seem to be associated with adverse outcome.

In the present study 51% of the patients were found to have epidural and/or paravertebral abscess formation. This high number can be explained by the fact that MRI and/or CT scans were systematically performed in all patients to establish the diagnosis of VO. All, but two of the patients (93%) with abscesses required surgery. This rate is much higher than reported in other studies [3,25]. All patients with abscess formation were treated according to our internal guidelines which are in line with published procedures [1]. This implies surgical drainage and/or debridement and is likely to have contributed to the high number of surgical intervention in case of abscess formation.

Open surgical decompression combined with intravenous antibiotic treatment has long been considered the cornerstone of management for spinal epidural abscess [26]. Following this management may be associated with a shorter duration of intravenous therapy in our study.

The aetiological microorganism could be isolated with blood cultures in 51% and with CT-guided or open biopsy in 75% of patients. *Staphylococcus aureus* and gram negative microorganisms were the most common causative microorganisms in our patient group. This is in line with previous studies where *Staphylococcus aureus* was the most common isolated organism followed by gram-negative bacilli, *E.coli* being the predominant agent [1,13,27]. Despite exclusion of endocarditis and spinal implants, as well as surgical site infections, 17% of the microorganisms detected were coagulase - negative staphylococci (CoNS) proven by bone biopsies. In these patients, biopsies were taken before empiric antibiotic therapy was initiated and CoNS-targeted antibiotic therapy led to resolution of symptoms. CoNS are common pathogens in cases of sternal osteomyelitis following median sternotomy [28] and are associated with intra-cardiac device related bacteraemia [29,30]. In contrast, they rarely cause osteomyelitis in the absence of bone devices or in patients without profound immunosuppression [31]. Our findings however indicate that CoNS might be more often found as the causative organisms in VO even without foreign bodies or endocarditis. We speculate that sclerosis of the bone in ageing patients may predispose to infections with CoNS.

In our study we had excellent outcome results. This might be due to the reason, that patients with endocarditis, immunodeficiency, vertebral implants and surgical site infection following spine surgery had been excluded. Latter mentioned patients tend to have more complications. E.g. recurrent bacteraemia in case of endocarditis was independently associated with relapse [18]. Another reason for the excellent outcome might be the high rate of drainage in case of abscesses. In the above mentioned study of Mc Henry et al. [18] surgical treatment resulted in recovery or improvement in 79% of patients.

A third reason for the excellent outcome is likely the fact that in every case an infectious disease specialist was involved and antibiotic therapy had continuously been adapted. In this respect the very low prevalence of MRSA may have helped the excellent outcome.

### Limitations and strengths

Our results may have a limited generalizability as the study was performed in a single centre. However, the study was conducted over an extended period of time with consistent results. Limited generalizability is also given due to the local epidemiology with a very low prevalence of methicillin-resistant *S. aureus* (MRSA) of 5-7% (data not shown). Due to frequently co-occuring resistance to rifampin in MRSA, one of the most important oral treatment options is futile in many instances. In our institution, we were able to switch to oral antibiotics in a large part of patients. A second limitation of the study is its retrospective study design; we cannot exclude a selection bias of patients who received a shorter intravenous antibiotic treatment. Selection bias may have occurred because physicians tend to switch earlier to an oral regimen if the patient has improved, so that switched patients may be those with better prognosis and/or more limited disease. On the other hand physicians may have had prolonged the intravenous therapy in patients which were not responding optimally to the therapy. Finally, the patient number did not allow for strong multivariate analyses, e.g. we could not define the level of C-reactive protein, above which intravenous therapy should be prolonged.

Our study has also strengths: First, the study covers a long time period with consistent results. Noteworthy, to the best of our knowledge, this is the first retrospective study of a homogenous patient collective (i.e. with primary vertebral osteomyelitis) after exclusion of patients with endocarditis, immunodeficiency, spinal implants and surgical site infection following spine surgery. Furthermore, the patient population was worked up in a meticulous way demonstrated by high rates of microbiological diagnoses and high rates of diagnosed abscesses.

## Conclusion

Our results suggest that switching to an oral antibiotic regimen after two weeks of intravenous therapy is safe in immunocompetent patients for primary non-implant vertebral osteomyelitis if epidural or paravertebral abscesses have been drained and if an oral antibiotic therapy with documented susceptibility, high bio-availability and bactericidal activity is available. Our results should be confirmed by a prospective randomized controlled trial.

## Competing interests

The authors declare that they have no competing interests.

## Authors’ contribution

BBF has made substantial contributions to conception of the study and interpretation of data and drafted the manuscript. LE has designed the study, performed statistical analysis, interpreted the data and helped to draft the manuscript. MK has collected the data and contributed in the conception of the study. RF has carried out the microbiological exams and contributed in data collection. MW has made substantial contribution to acquisition of data and has revised the manuscript critically. SS has performed the surgical procedures and revised the manuscript. AW has made substantial contribution in data interpretation. MB participated in the design and coordination of the study helped to draft the manuscript. All authors read and approved the final manuscript.

## Pre-publication history

The pre-publication history for this paper can be accessed here:

http://www.biomedcentral.com/1471-2334/14/226/prepub
